# Impact of co-existence of PMQR genes and QRDR mutations on fluoroquinolones resistance in Enterobacteriaceae strains isolated from community and hospital acquired UTIs

**DOI:** 10.1186/s12879-019-4606-y

**Published:** 2019-11-21

**Authors:** Dalia Nabil Kotb, Wafaa Khairy Mahdy, Mahmoud Shokry Mahmoud, Rasha M. M. Khairy

**Affiliations:** Department of Microbiology and Immunology, Faculty of Medicine, Minia University, Minia, 61511 Egypt

**Keywords:** *Enterobacteriaceae*, Fluoroquinolones, Plasmid-mediated quinolone resistance, Quinolone resistance-determining regions (QRDRs)

## Abstract

**Background:**

Fluoroquinolones are commonly recommended as treatment for urinary tract infections (UTIs). The development of resistance to these agents, particularly in gram-negative microorganisms complicates treatment of infections caused by these organisms. This study aimed to investigate antimicrobial resistance of different *Enterobacteriaceae* species isolated from hospital- acquired and community-acquired UTIs against fluoroquinolones and correlate its levels with the existing genetic mechanisms of resistance.

**Methods:**

A total of 440 *Enterobacteriaceae* isolates recovered from UTIs were tested for antimicrobial susceptibility. Plasmid-mediated quinolone resistance (PMQR) genes and mutations in the quinolone resistance-determining regions (QRDRs) of *gyrA* and *parC* genes were examined in quinolone-resistant strains.

**Results:**

About (32.5%) of isolates were resistant to quinolones and (20.5%) were resistant to fluoroquinolones. All isolates with high and intermediate resistance phenotypes harbored one or more PMQR genes. Q*nrB* was the most frequent gene (62.9%) of resistant isolates. Co-carriage of 2 PMQR genes was detected in isolates (46.9%) with high resistance to ciprofloxacin (CIP) (MICs > 128 μg/mL), while co-carriage of 3 PMQR genes was detected in (6.3%) of resistant isolates (MICs > 512 μg/mL). Carriage of one gene only was detected in intermediate resistance isolates (MICs of CIP = 1.5–2 μg/mL). Neither *qnrA* nor *qnrC* genes were detected. The mutation at code 83 of *gyrA* was the most frequent followed by Ser80-Ile in p*arC* gene, while Asp-87 Asn mutation of *gyr*A gene was the least, where it was detected only in high resistant *E. coli* isolates (MIC ≥128 μg/mL). A double mutation in *gyrA* (Lys154Arg and Ser171Ala) was observed in high FQs resistant isolates (MIC of CIP < 128 μg/mL).

**Conclusion:**

FQs resistance is caused by interact between PMQR genes and mutations in both *gyrA* and *parC* genes while a mutation in one gene only can explain quinolone resistance. Accumulation of PMQR genes and QRDR mutations confers high resistance to FQs.

## Background

Urinary tract infections (UTIs) are common infectious diseases in both hospital- acquired UTI (HAUTI) and community-acquired UTI (CA-UTI) [1, 2]. UTI can be diagnosed by at least one of the following clinical symptoms or signs; temperature ≥ 38 °C, suprapubic pain, costovertebral angle pain, urinary urgency, dysuria or frequency. A quantitative urine culture with bacterial counts ≥10^5^ colony forming units per mL (CFU/mL) is essential for the diagnosis of UTI [3]. UTIs may be caused by gram-negative, gram-positive bacteria or by fungi. *E. coli* is the most common cause followed by *Klebsiella* and other *Enterobacteriaceae* in both CA-UTI [4] and hospital- acquired UTI (HAUTI) [5]. Fluoroquinolones (FQs) have been considered a highly effective treatment of UTIs. However, the development of resistance to these agents especially by gram-negative microorganisms complicates the treatment of infections caused by these organisms [6]. FQs resistance is mainly caused by spontaneous mutations in the quinolone resistance determining regions (QRDR) of *gyr* A and *par* C genes, either *gyr* A or *par* C, or both genes [7]. However, in the past few decades, plasmid-mediated quinolone resistance (PMQR) has been increasingly reported in *Enterobacteriaceae* species all over the world [8]. The co-existence of mutations in QRDR and PMQR genes carriage can occur together in *Enterobacteriaceae* species. Moreover, the presence of PMQR determinants may promote QRDR mutations increasing the FQs resistance rates [9]. Some studies in Egypt have previously investigated the FQs resistance [10, 11], but the study of contribution of various mechanisms of resistance in different *Enterobacteriaceae* species in immunocompetent patients was not addressed. Accordingly, the aim of the current study was to investigate antimicrobial resistance of different *Enterobacteriaceae* species isolated from CA-UTI and HAUTI against FQs and correlate its levels with the existing genetic mechanisms of quinolones (Qs) resistance.

## Methods

### Study design

This is a cross-sectional study was conducted in Minia university hospitals, Egypt, from July 2016 to March 2017. A total of 705 patients with suggested UTI (presented with one or more of UTI symptoms;(Fever ≥38 °C, dysuria, increased frequency and suprapubic pain) were included in the study. Urine samples with positive pyuria and urine cultures with a colony count for a single bacterial species ≥10^5^ CFU/mL were only included. The study populations were adults (> 18 years). About 32.2% of participants were males and 67.8% were females. The study included 418 outpatients (attended at outpatient’s clinics seeking the treatment) and 286 inpatients (developed their clinical symptoms after 48 h of admission). This study was carried out according to the principles of the declaration of Helsinki. The study was approved by the Medical Ethics Committee of Minia university Hospital, Egypt. As the study used anonymous clinical data, the patients were not required to give informed consent for the study (code: 45 A at 2/5/2016).

### Bacterial strains

Urine specimens were collected from symptomatic patients in sterile screw capped containers that were transported within 2 h of collection to bacteriology laboratory in an ice box and processed at once. Well-mixed uncentrifuged urine specimens were streaked by semi-quantitative streaking method onto UTI chrome agar (CHROMagar™ Orientation, paris, France) and by calibrated loop technique on MacConkey and blood agars [12]. After overnight incubation at 37 °C, isolated uropathogens were further identified according to their phenotypic criteria based on gram staining, cultural characters and biochemical testing including indole, urease, citrate and sugar fermentation tests [12]. Confirmed *Enterobacterecieae* strains were kept in trypticase soy broth with sterilized 15% glycerol at − 20 °C. A total of 440 non-repetitive *Enterobacterecieae* isolates were recovered from urine samples.

### Antimicrobial susceptibility testing

According to CLSI guidelines [13], disk diffusion method was used to determine antimicrobial susceptibility of the *Enterobacteriaceae* isolates to different antibiotics; amoxicillin/clavulanic acid (AMC) 30 μg, ceftriaxone (CRO) 30 μg, ceftazidime (CAZ) 30 μg, imipenem (IPM) 10 μg, amikacin (AK) 30 μg, sulphamethoxazole/ trimethoprim (SXT) 25 μg, and nitrofurantoin (F) 300 μg. Also 4 different antibiotics discs for quinolone (Q) and fluoroquinolones (FQs) resistance were used; nalidixic acid (NA) 30 μg, ciprofloxacin (CIP) 5 μg, norfloxacin (NOR) 10 μg and ofloxacin (OFX) 5 μg (Thermo Scientific™ Oxoid, UK). Minimum inhibitory concentration (MIC) of ciprofloxacin (CIP) was determined by two methods; MIC strips (E test) which graded from 0.002 to 32 μg/mL and agar dilution method. MIC strips (E test) (Liofilchem s.r.l, Italy) were placed on surfaces of inoculated Mueller-Hinton agar plates as explained previously [12]. MIC determination by agar dilution method was performed according to CLSI guidelines (CLSI, 2015 and CLSI*, 2015), where ten concentrations (4–2048 μg/mL) of CIP were prepared (each in a single agar plate) [14, 15]. The results of disk diffusion assay as well as MIC were interpreted according to CLSI guidelines [13].

### DNA extraction and PCR amplification

DNA was extracted using GeneJET genomic DNA purification kit (Thermosceintific, USA) according to the manufacturer’s instructions. PMQR genes; *qnrA, qnrB* and *qnrS* were tested by a multiplex PCR reaction using specific primers (Table 1). PCR was performed in a 25 μL reaction mixture containing 5 μL of purified DNA (approximately 500 ng/휇L), 12.5 μL of Platinum® multiplex PCR master Mix (Applied Biosystems™, USA), 0.8 휇L (8 pmol) of each primer and 2.7 μL of nuclease free water. Single PCR reactions were used for amplification of each of PMQR genes (*qnrC* and *qepA)* and QRDRs of *gyrA* and *parC* genes using specific primers (Table 1). Each single PCR reaction was performed in a 25 μL reaction mixture containing 300 ng/휇L of DNA, 12.5 μL of AmpliTaq Gold® 360 master mix (Applied Biosystems™, USA), 1 μL (10 pmol) of each primer and 7.5 μL of nuclease free water. The primers sequences, annealing temperature, and size of amplified fragments for the studied genes are shown in Table 1 [16–20]. PCR products were resolved on 1% agarose gel with ethidium bromide dye and the gel was visualized under a UV transilluminator (Biometra Goettingen, Germany).

**Table 1:** PCR primers used in the current study

Primer name	Primer sequence (5^/^ to 3^/^)	PCR product size (bp)	Annealing Temperature	Ref
*qnrA* m-F	AGAGGATTTCTCACGCCAGG	580	54 °C	[16]
*qnrA* m-R	TGCCAGGCACAGATCTTGAC			
*qnrB* m-F	GGMATHGAAATTCGCCACTG	264	54 °C	[16]
*qnrB* m-R	TTTGCYGYYCGCCAGTCGAA			
*qnrS* m-F	GCAAGTTCATTGAACAGGGT	428	54 °C	[16]
*qnrS* m-R	TCTAAACCGTCGAGTTCGGCG			
*qnrC*-F	GGGTTGTACATTTATTGAATC	447	50 °C	[17]
*qnrC*-R	TCCACTTTACGAGGTTCT			
*qepA*-GF	ACATCTACGGCTTCTTCGTCG	502	55 °C	[18]
*qepA*-GR	AACTGCTTGAGCCCGTAGATC			
*parC-*F	ATG TAC GTG ATC ATG GAC CG	300	55 °C	[19]
*parC*-R	ATT CGG TGT AAC GCA TCG CC			
*gyrA*-F	AAA TCT GCC CGT GTC GTT GGT	343	55 °C	[20]
*gyrA*-R	GCC ATA CCT ACG GCG ATA CC			

### Sequencing of PCR products

Sequencing was carried out using an automated DNA sequencer and data collection software from Applied Biosystems, USA. Translated nucleotide sequences of QRDR in *gyrA* and *parC* genes were compared with corresponding reference protein sequences using BLAST software of NCBI; National Center for Biotechnology Information (http://www.ncbi.nlm.nih.gov/blast). *GyrA* accession number used for *E. coli* was WP_074153749.1, for *Klebsiella* spp. was WP_075874334.1 and for *Citrobacter* spp. was WP_044266198.1. Accession number for *parC* was AML00471.1.

### Statistical analysis

Statistical analysis of demographic, clinical and laboratory data of study subjects was performed using SPSS for windows version 19.0 (IBM, USA).

## Results

### Prevalence of *Enterobacteriaceae* strains among UTIs

Four hundred and forty isolates belonging to *Enterobacteriaceae* family were recovered from UTI patients with a percentage of (440/705, 62.4%). Two hundred and nine (209/286, 73.1%) were from inpatients and (231/418, 55.1%) were from outpatients. *E. coli* was the most frequent pathogen (281/440 (63.9%), followed by *Klebsiella pneumoniae* (*K. pneumoniae)* (81/440, 18.4%), *Citrobacter* spp. (47/440, 10.7%), *Proteus* spp. (20/440, 4.5%) and lastly *Enterobacter* spp. (11/440, 2.5%), (Table 2).

**Table 2:** prevalence of different species of *Enterobacteriaceae* isolated from UTIs

Organism	Frequency						*P* value
	Total		Outpatients		Inpatients		
	N	%	N	%	N	%	
*E.coli*	281	63.9%	147	63.6%	134	64.1%	0.0001
*K. pneumoniae*	81	18.4%	40	17.3%	41	19.6%	
*Citrobacter* spp.	47	10.7%	17	7.4%	30	14.4%	
*Enterobacter* spp.	11	2.5%	9	3.9%	2	1.0%	
*Proteus* spp.	20	4.5%	18	7.8%	2	1.0%	
Total	440	100%	231	100%	209	100%	

*UTIs* Urinary tract infections

### Antimicrobial susceptibility profiles of *Enterobacteriaceae* isolates

Of 440 *Enterobactericeae* clinical isolates tested for antimicrobial susceptibility, the highest rates of resistance were observed against SXT (253/440, 57.5%), CRO (217/440, 49.3%) and AMC (159/440, 36.1%). while the highest susceptibility rate was found to IMP (440/440, 100%). Percentages of resistant isolates to tested antimicrobial agents are summarized in Table 3.

**Table 3:** Patterns of antimicrobial resistance of *Enterobacteriaceae* species isolated from UTIs

Antibiotic	AMC		CTZ		CRO		IMP		AK		SXT		NA		CIP		NOR		OFX		F	
organism	N	%	N	%	N	%	N	%	N	%	N	%	N	%	N	%	N	%	N	%	N	%
*E.coli*	99	35.2	97	34.5	159	56.6	0	.0	63	22.4	182	64.8	104	37	54	19.2	54	19.2	54	19.2	69	24.6
*K. pneumoniae*	17	21.0	13	16.0	16	19.8	0	.0	9	11.1	29	35.8	11	13.6	9	11.1	9	11.1	9	11.1	12	14.8
*Citrobacter* spp*.*	31	66.0	20	42.6	30	63.8	0	.0	10	21.3	22	46.8	17	36.2	17	15.6	17	15.6	17	15.6	19	40.4
*Enterobacter* spp*.*	1	9.1	1	9.1	1	9.1	0	.0	1	9.1	2	18.2	1	9.1	0	0	0	0	0	0	0	0.0
*Proteus* spp.	11	55.0	11	55.0	11	55.0	0	.0	1	5.0	18	90.0	10	50	10	9.2	10	9.2	10	9.2	10	50
Total	159	36.1	142	32.3	217	49.3	0	.0	84	19.1	253	57.5	143	32.5	90	20.5	90	20.5	90	20.5	110	25

*AMC* Amoxicillin Clavulanic acid, *CTZ* Ceftazidime, *CRO* Ceftriaxone, *IMP* Imipenem, *AK* Amikacin, *SXT* Sulphamethoxazole-Trimethoprim, *NA* Nalidixic acid, *CIP* Ciprofloxacin, *NOR* Norfloxacin, *OFX* Ofloxacin, *F* Nitrofurantoin

### Quinolone (Q) and fluoroquinolones (FQs) susceptibility

A total of (143/440, 32.5%) *Enterobacteriaceae* isolates were resistant to one or more of the tested (Q and FQs). Out of them (90/440, 20.5%) were resistant to FQs. These isolates could be categorized into three phenotypes; high resistance phenotype which included 67 isolates that were highly resistant to all tested Q and FQs with MIC of CIP > 32 μg/mL, intermediate resistance phenotype which included 23 isolates with intermediate resistance to all tested Q and FQ with MIC of CIP =1–2 μg/mL. FQs susceptible phenotype which included 53 isolates that were resistant to NA only (Q) and susceptible to all tested FQs with MIC of CIP ≤ 1 μg/mL.

### Prevalence of PMQRs

Of the 143 Q resistant isolates, 90 isolates (62.9%) harbored at least one PMQR gene (54 *E. coli*, 17 *Citrobacter*, 9 *K. pneumoniae* and 10 *Proteus).* The most frequent PMQR gene was *qnrB*, which was detected in (90/143, 62.9%) of Q and FQs resistant isolates and in (90/90, 100%) of FQs resistant isolates. Q*nrS* gene was detected in (67/143, 46.9%) of Q and FQs resistant isolates and in (67/90, 74.4%) of FQs resistant isolates (Additional file 1: Figure S1). Neither *qnrA* nor *qnrC* were detected in the studied isolates. *qepA* gene was detected in (9/143, 6.3%) of Q and FQs resistant isolates and in (9/90, 10%) of FQs resistant isolates. (Additional file 2: Figure S2) (Table 4).

**Table 4:** distribution of PMQR and QRDRs among different FQs resistance phenotypes of *Enterobacteriaceae* isolated from UTIs

Species	Phenotype	Resistant to Quinolones	PMQR genes	MIC of CIP (μg/mL)	Number	gyrA alterations	ParC alterations
*E.coli*	FQ Susceptible	NA only	No genes detected	0.5	31	Ser83Leu	No mutation
				0.25	19	Ile155Phe	Ser80Ile
	Intermediate resistance	NA, CIP, NOR, OFX	*qnrB*	1.5	7	His80Met Gly81Ala Ser83Leu	Met118Trp Arg119Val
				2	13	Ser83Leu	No mutation
	High resistance	NA, CIP, NOR, OFX	*qnrB, qnrS*	128	28	Ser83Tyr Lys154Arg Ser171Ala Ile174Thr Ala175Val	Ser80Ile
	High resistance	NA, CIP, NOR, OFX	*qnrB, qnrS, qepA*	128, 256, 512	6	Asp87Asn	Ser80Ile
*K. pneumoniae*	FQ Susceptible	NA only	No genes detected	0.5	2	Lys154Arg Ala171Ser	No mutation
	Intermediate resistance	NA, CIP, NOR, OFX	*qnrB*	2	2	No mutation	Ser80Ile
	High resistance	NA, CIP, NOR, OFX	*qnrB, qnrS*	128	3	Ser83Tyr	Ser80Ile Met118Ile
				512	4	Ser83Tyr Ala175Arg Val176Leu	Ser80Ile Met118Ile
*Citrobacter* spp.	Intermediate resistance	NA, CIP, NOR, OFX	*qnrB*	1.5	1	ND	ND
	High resistance	NA, CIP, NOR, OFX	*qnrB, qnrS*	256	15	Ser83Ile Lys154Arg Ser171Ala	Ser80Ile
	High resistance	NA, CIP, NOR, OFX	*qnrB, qnrS, qepA*	512	1		
*Enterobacter* spp.	FQ susceptible	NA only	No genes detected	0.125	1	ND	ND
*Proteus* spp.	High resistance	NA, CIP, NOR, OFX	*qnrB, qnrS*	125–512	8	ND	ND
	High resistance	NA, CIP, NOR, OFX	*qnrB, qnrS, qepA*		2	ND	ND

*PMQR* Plasmid-mediated quinolone resistance, *QRDR* Quinolone resistance determining regions, *CIP* Ciprofloxacin, *ND* Not determined

### QRDR mutations in *gyrA* and *parC* genes

**Fig. 1:**
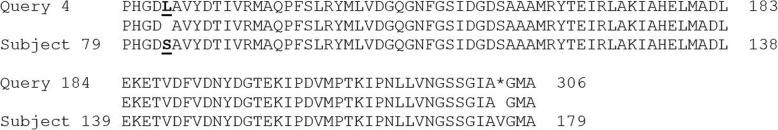
Alteration in *gyr A* (codon 83). Nucleotide sequence of a *gyrA* region of the *E. coli* FQs-susceptible, WP_074153749.1 DNA gyrase subunit A (*E. coli*). Alteration in (codon 83)

**Fig. 2:**
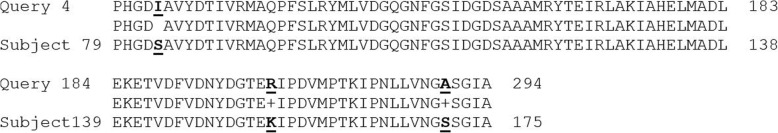
Alteration in *gyr A* (codon 83, 154, 171). Nucleotide sequence of a *gyrA* region of the *Citrobacter* spp. high resistance isolates, WP_044266198.1 DNA gyrase subunit A (*Citrobacter)*. Alteration in (codon 83, 154, 171)

**Fig. 3:**
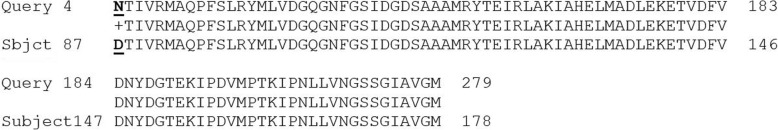
Alteration in *gyr A* (codon 87). Nucleotide sequence of a *gyrA* region of the *E. coli* high resistance isolates, WP_074153749.1 DNA gyrase subunit A (*E. coli*) Alteration in (codon 87)

**Fig. 4:**
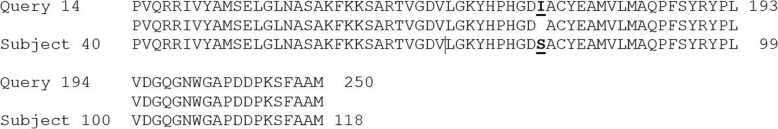
Alteration in *par C* (codon 83). Nucleotide sequence of a *parC* region of the *E. coli* high resistance isolates, Reference Sequence Strain. AML00471.1 DNA topoisomerase IV. Alteration in (codon 80)

Q and FQs resistant isolates were studied by PCR and subsequent sequencing of QRDR of their *gyrA* and *parC* genes. Mutation at codon 83 of *gyrA* was detected in (102/143, 71.3%) of Q resistant isolates; 51 of them belong to high resistance phenotype, 20 in intermediate phenotype and 31 belong to the FQs susceptible phenotype (resistant to NA only) (Figs. 1 and 2). Mutation at codon 87 (Asp87Asn) of *gyrA* was detected in high resistance phenotype only (6 isolates) (Fig. 3). Three types of amino acid changes resulted from mutation at codon 83 of *gyrA* protein; change from serine to leucine in *E. coli*, from serine to tyrosine which was detected in *E. coli* and *Klebsiella* and from serine to isoleucine in *Citrobacter* strains. Mutation at position 80 of *parC* gene was detected in (78/143, 54.5%) of isolates (Fig. 4); 57 of them belong to high resistance phenotype, 2 isolates (intermediate resistance) and 19 isolates (FQs susceptible phenotype). Isolates’ identification, phenotypes, PMQR genes distribution and detected mutations are summarized in (Table 4).

### Correlation between phenotyping and genotyping of resistant isolates

All isolates with high resistance phenotype (67) harbor *QnrB* and q*nrS* genes. Q*epA* gene was detected in only 9 isolates with high resistance phenotype. Isolates with intermediate resistance phenotype (23) carry *qnrB* gene only. None of PMQR genes was found in isolates with FQs susceptible isolates (resistant to NA only). Detection of PMQR genes (*qnrB* and *qnrS*) was strongly correlated with FQs resistance levels in resistant isolates with a correlation coefficient equal to 0.8 (r = 0.8) and 0.9 (r = 0.8) respectively which was highly significant (*p* value; 0.0001) for both genes as well as *qepA* gene with a correlation coefficient equal to 0.25 (r = 0.25) which was considered fair but significant (*p* value; 0.006). Detection of specific mutations in *gyrA* and *parC* genes was not correlated with susceptibility pattern to tested Q and FQs as these mutations were found in all phenotypes (Table 4).

## Discussion

UTIs are common bacterial infections in hospital settings and community [21]. In the current study, 440 *Enterobacteriaceae* isolates were isolated from UTIs with a percentage of (440/705, 62.4%). The isolation rates from inpatients and outpatients were (73.1%) and (55.1%) respectively. A higher frequency of isolation (86%) was recorded previously in Asia-Pacific region [22]. The frequency of isolation from outpatients agrees with a previous report in Ethiopia (57.75%) [23], and disagrees with another report in Korea (89%) [4]. *E. coli* was the most frequent followed by *K. pneumoniae*, *Citrobacter* spp.*, Proteus* spp. and lastly *Enterobacter* spp. These findings agree with several previous studies [24, 25]. In spite of similarity with these reports, *Citrobacter* spp*.* isolation rate in the current study remains the highest. The highest antimicrobial resistance rates were recorded in the present study against SXT with a percentage of (57.5%) followed by CRO (49.3%), however, none of the isolates were resistant to imipenem. These findings agree with several previous studies [4, 5]. High resistance to CRO may be caused by extensive use in the locality. NA has been used for treatment of UTIs for more than five decades [26] so the resistance to NA is expected to be higher than to FQs. In the current study, NA has a resistance rate of (143/440, 32.5%), however higher rates were reported previously in several studies [23, 25, 27]. The fact that NA is not an empiric treatment of UTI in Egypt may be the cause of this difference. FQs have an overall resistance rate of (90/440, 20.5%). Near results were reported in Korea (24.8%) [4], while a higher resistance rate (54.9%) was recorded in Asian countries [28]. With the analysis of PMQR genes (*qnr* genes and *qepA* gene), the most frequent gene was *qnrB*, which was detected in (62.9%) of Q and FQs resistant isolates, and in (100%) of FQs resistant isolates while *qnrS* gene was detected in (46.9%) of Q and FQs resistant isolates and in (74.4%) of FQs resistant isolates. In other studies on Egyptian population, *qnrB* was the most prevalent *qnr* gene among *K. pneumoniae* isolates as denoted by El-Badawy et al., 2017 which agree with our results [29], while others reported that *qnrS* was the most prevalent gene among gram negative bacilli isolated from different clinical settings [10, 11]. *QnrB* and *qnrS* genes detection rate in our study was higher than that reported in several previous studies [27, 30]. Neither *qnrA* nor *qnrC* were detected at all, but *qepA* gene was detected in (10%) of FQs resistant isolates. In the same context with us, several studies also could not detect *qnrA* gene among *Enterobacteriaceae* isolates [27, 30, 31]. However, Szabó et al., 2018 could detect *qnrA* in their isolates but could not detect neither *qnrC* nor *qepA* genes [32]. The difference between our results and others may be caused by geographical distribution of *qnr* genes, type of clinical isolates or the used methods of detection. With considering the analysis of the QRDRs of *gyrA* and *parC* genes, this study reported that the mutation at position 83 of *gyrA* was the most frequent in Q and FQs resistant strains. Three types of amino acid changes resulted from this mutation. Change from serine to leucine in *E. coli* strains which was also reported by many reports [33, 34] and change from serine to tyrosine which was detected in *K. pneumoniae* and *E. coli* and was also reported previously [35]. The third change (from serine to isoleucine) was detected in *Citrobacter* strains and was reported in several studies [36, 37]. A double concomitant mutation in *gyrA* A (Lys154Arg and Ser171Ala) was observed in high FQs resistant C*itrobacter* spp. isolates with MIC of CIP ≥ 256 μg/mL and *E. coli* isolates with MIC of CIP ≥128 μg/mL. These mutations were reported previously in few studies [35, 38]. Asp-87 Asn mutation of *gyr*A gene was the least frequent mutation, where it was detected only in high resistant *E. coli* isolates with MIC ≥128 μg/mL. Therefor the mutation at position 87 of *gyrA* seemed to contribute to high resistance to FQs, while the mutation at position 83 could contribute to both Q and FQs resistance. This suggestion agrees with other results [34, 39]. Ser80Ile mutation of *parC* gene was frequent in both Q and FQs resistant strains that agrees with other studies [40, 41]. Single mutation in either gene occurred only in isolates with MIC of CIP ≤ 2 μg/mL, so the presence of double mutation in *gyrA* and *parC* genes seemed to be associated with high levels of resistance to FQs. These findings agree with other reports suggested that high levels of FQs resistance appeared to happen as a result of gradual accumulation of QRDR mutations [38, 42]. All isolates with high and intermediate resistance phenotypes harbored one or more PMQR gene, interestingly, isolates with FQs susceptible phenotype (resistant to NA only) harbored none of the tested PMQR genes. This agrees with Rodrı’guez-Martı’nez et al., 2016, who stated that resistance to NA only is not enough to suggest presence of PMQR genes [43], while Szabó and his colleagues could found PMQR genes among susceptible or low-level resistance to ciprofloxacin with (MIC = 0.06–1 mg/L) isolates [32]. Our study also agrees with Piekarska et al., 2015, who stated that combination of both PMQR genes and mutations in QRDRs of *gyrA* and *parC* contributes to high resistance to FQs [9].

## Conclusion

In the current study *Enterobactericeae* remain the most common cause of UTIs. The resistance rate of Q is (32.5%), while the resistance to Q and FQs is (20.5%) among *Enterobactericeae* isolates. At least one of PMQR gene was detected in FQs resistant isolates. The most frequent gene was *qnrB*, which was detected in (62.9%) of Q resistant isolates followed by *qnrS* gene which was detected with a percentage of (46.9%). The co-existence of 2 PMQR genes in the same isolate was observed in (46.9%) of resistant isolates, while co-existence of 3 PMQR genes was reported in (6.3%). The presence of at least two PMQR genes together with simultaneous QRDR mutations in each of *gyrA* and *parC* genes can describe the mechanism of resistance in high resistance phenotype (highly resistant to all tested Q and FQs), while presence of at least one PMQR gene together with one QRDR mutation at either genes could be the cause of resistance in isolates with intermediate resistance phenotype (intermediate resistance to all tested Q and FQs). Presence of mutation in only one QRDR regions of *gyrA* or *parC* genes could be the cause of resistance to NA only. To our knowledge, the current study is the largest study that reported molecular epidemiology of quinolones resistance in different *Enterobacteriaceae* species in the study area and could suggest a phenotypic algorithm to describe genetic mechanisms of quinolone resistance.

## Supplementary information

**Additional file 1: Figure S1.** Agarose gel electrophoresis (1%) for separation of multiplex PCR products; M is molecular size marker (100 bp ladder), lanes: 1, 2, 3 are positive for *qnrB* and *qnrS*, 4, 5 are positive for *qnrB*. The size of PCR products (in base pairs) is indicated on the right.

**Additional file 2: Figure S2.** Agarose gel electrophoresis (1%) for separation of PCR products of *qepA*; M is molecular size marker (100 bp ladder), lanes: 1, 2, 3, 4, 5, 6, 9 are positive for *qepA,* 7, 8 are negative for *qepA* gene. The size of PCR product (in base pairs) is indicated on the right.

## Data Availability

All data generated or analyzed during this study are included in this article [and its supplementary information files (Additional file 1: Figure S1 and Additional file 2: Figure S2)].

## Abbreviations

CA-UTI: Community-acquired urinary tract infection
CIP: Ciprofloxacin
CRO: Ceftriaxone
FQs: Fluoroquinolones
HAUTI: Healthcare-associated urinary tract infection
MIC: Minimum inhibitory concentration
PMQR: Plasmid-mediated quinolone resistance
QRDRs: Quinolone resistance-determining regions
Q: Qunolone
UTI: Urinary tract infection
