# Supplementary figures and images for: Impact of co-existence of PMQR genes and QRDR mutations on fluoroquinolones resistance in Enterobacteriaceae strains isolated from community and hospital acquired UTIs
Source: BMC Infect Dis. 2019 Nov 21;19:979. doi: 10.1186/s12879-019-4606-y (PMC6868749; doi:10.1186/s12879-019-4606-y)

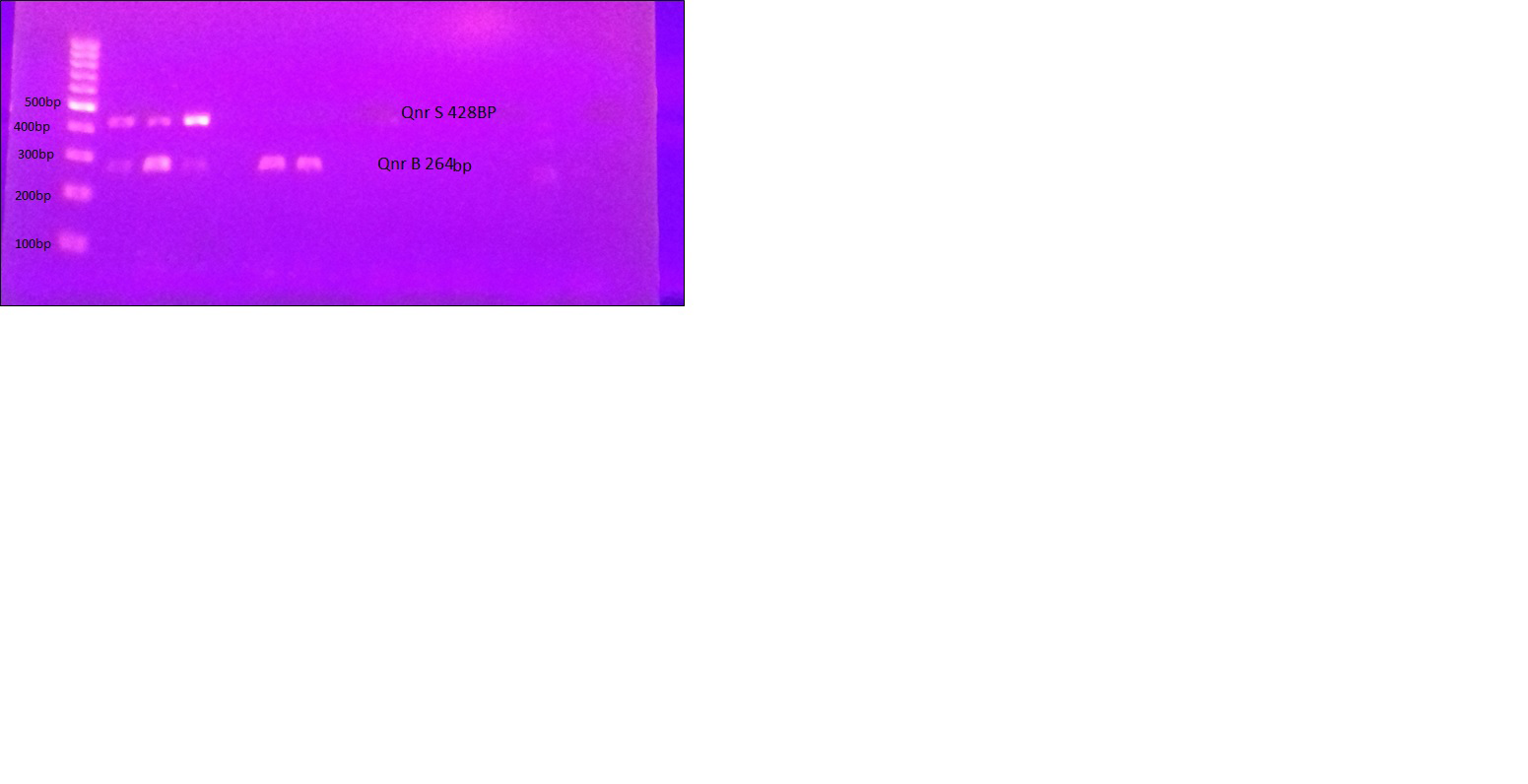

Supplement: Supplementary file 1 — Additional file 1: Figure S1. Agarose gel electrophoresis (1%) for separation of multiplex PCR products; M is molecular size marker (100 bp ladder), lanes: 1, 2, 3 are positive for qnrB and qnrS, 4, 5 are positive for qnrB. The size of PCR products (in base pairs) is indicated on the right. [file 12879_2019_4606_MOESM1_ESM.tif]

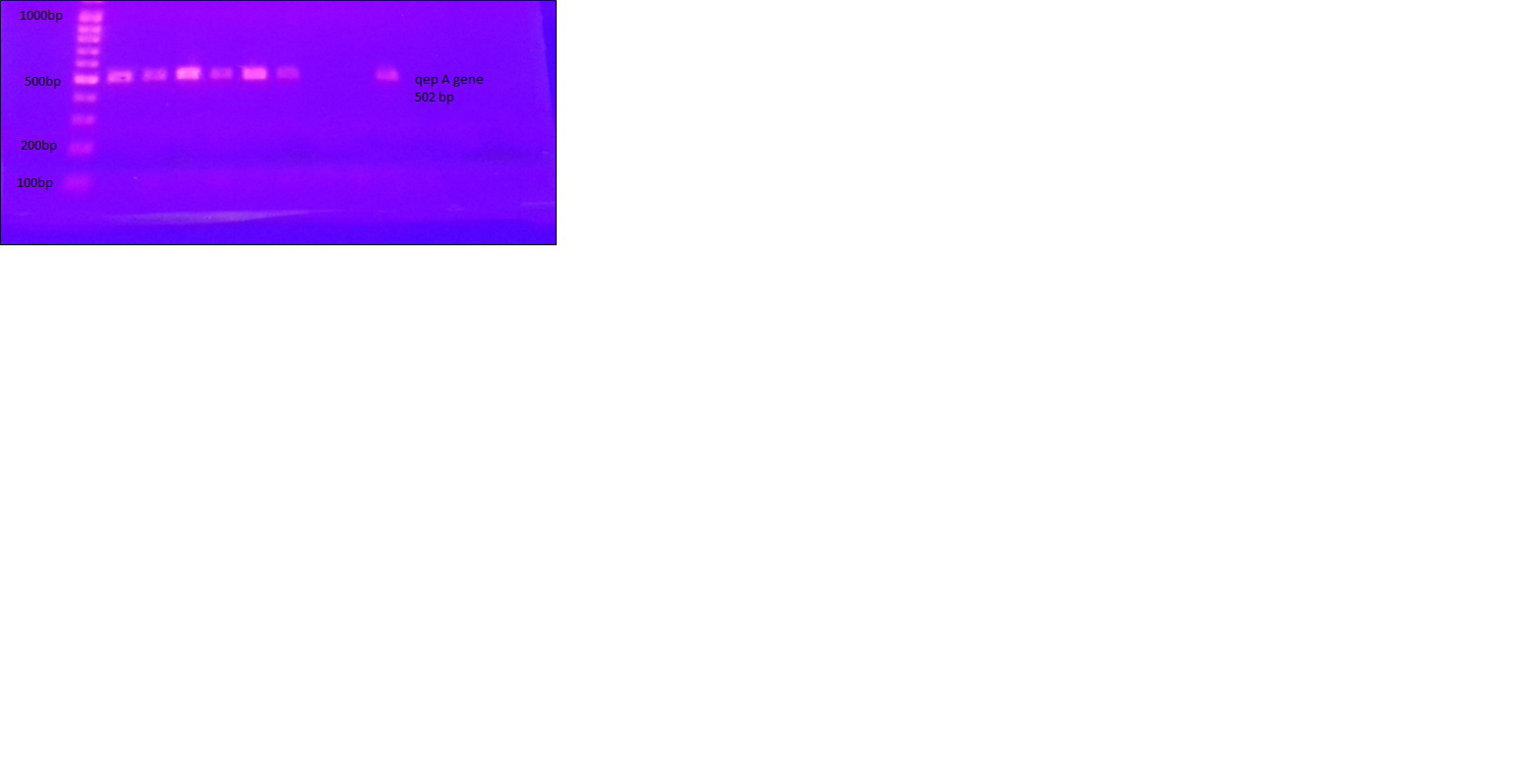

Supplement: Supplementary file 2 — Additional file 2: Figure S2. Agarose gel electrophoresis (1%) for separation of PCR products of qepA; M is molecular size marker (100 bp ladder), lanes: 1, 2, 3, 4, 5, 6, 9 are positive for qepA, 7, 8 are negative for qepA gene. The size of PCR product (in base pairs) is indicated on the right. [file 12879_2019_4606_MOESM2_ESM.tif]
